# A novel neuroprotective mechanism of selegiline by suppressing the pro-apoptotic activity of protein disulfide isomerase

**DOI:** 10.1186/s43556-025-00255-w

**Published:** 2025-03-17

**Authors:** Yuting Xie, Bing Chen, Piao Luo, Jingnan Huang, Jigang Wang, Jichao Sun, Zhen Liang

**Affiliations:** 1 Department of Geriatrics, Guangdong Provincial Clinical Research Center for Geriatrics, Shenzhen Clinical Research Center for Geriatrics, Shenzhen People’s Hospital (The First Affiliated Hospital, Southern University of Science and Technology; The Second Clinical Medical College, Jinan University), Shenzhen, Guangdong China; 2 School of Medicine, Southern University of Science and Technology, Shenzhen, Guangdong China; 3 Department of Geriatrics, Peking University Shenzhen Hospital, Shenzhen, Guangdong China

Dear Editor

Parkinson’s disease (PD) is a chronic neurodegenerative disorder that remains incurable, due to its complexity and the incomplete understanding of its underlying mechanisms. Current management primarily relies on pharmacological treatments, such as levodopa, to increase brain dopamine levels. To prolong the therapeutic effects of dopamine, MAO-B inhibitors are used in conjunction with levodopa to slow dopamine metabolism and enhance treatment efficacy. Selegiline, the first FDA-approved MAO-B inhibitor for PD, alleviates symptoms by inhibiting the metabolism of dopamine and reducing neurotoxic metabolites. Beyond its role in MAO-B inhibition, selegiline exhibits neuroprotective effects, potentially through anti-apoptotic mechanisms. Recent studies have shown that selegiline can prevent mitochondria-dependent apoptosis [1], whereas the specific molecular targets for such effect remain elusive.

In the present work, we utilized selegiline’s unique propargylamine moiety for click chemistry to screen and identify its potential protein targets in the mouse dopaminergic neuronal MN9D cell line, using activity-based protein profiling (ABPP) technology (Fig. 1a, left). The labeling efficiency of selegiline was confirmed to be dose-dependent using the copper-catalyzed azide–alkyne cycloaddition reaction (CuAAC) with TAMRA-azide (Supplementary Fig. 1a). We then performed the chemical proteomics experiment to identify targets of selegiline. Proteins with a threshold standard of fold change ≥ 2 and *p* value < 0.05 were considered as potential targets of selegiline (Fig. 1a, right). Remarkably, a set of proteins belonging to the protein disulfide isomerase (PDI) family, including PDI, PDIA3, PDIA4 and PDIA6, emerged as top hits among selegiline’s targets (Fig. 1a, right). We selected PDI (P4HB) as the representative hit for further examination, given its top ranking in fold change and its role as the founding and most versatile member of the PDI family. In situ pull-down assay revealed that PDI was successfully isolated by selegiline (Fig. 1b, left). To further investigate the interaction between PDI and selegiline, we employed cellular thermal shift assay (CETSA), a biophysical tool that characterizes ligand–protein engagement by measuring ligand-induced changes in thermal stability. Our results showed that selegiline significantly enhanced PDI's thermal stability compared to DMSO-treated controls (Fig. 1b, right; Supplementary Fig. 1b). Next, we purified recombinant PDI protein and treated it with varying concentrations of selegiline (0–160 µM), followed by TAMRA-azide labeling via CuAAC. The Fluorescence intensity of the PDI protein increased linearly with the selegiline concentration (Fig. 1c, upper left). Notably, the active cysteine alkylating agent iodoacetamide (IAA) significantly competed with selegiline labeling (Fig. 1c, lower left), suggesting that selegiline binds to the reactive cysteine residues in PDI. To evaluate the binding affinity of selegiline to PDI, we performed surface plasmon resonance (SPR) analysis, revealing a dissociation constant (KD) of 96 µM (Supplementary Fig. 1c). We then conducted tandem mass spectrometry to identify the specific PDI residues modified by selegiline. Our results revealed that selegiline covalently binds to Cys56 (Fig. 1c, right), Ser32 and Lys207 (Supplementary Fig. 1d). Molecular docking analysis corroborated these findings (Data not shown). To further validate the identified binding sites, we introduced point mutations (S32A, C56A and K207A) into PDI. As expected, the labeling intensity decreased for single, double (S32A and C56A, 2A) and triple (S32A, C56A and K207A, 3A) mutants, with the 3A mutation displayed the most significant reduction (Supplementary Fig. 1e).

**Fig. 1:**
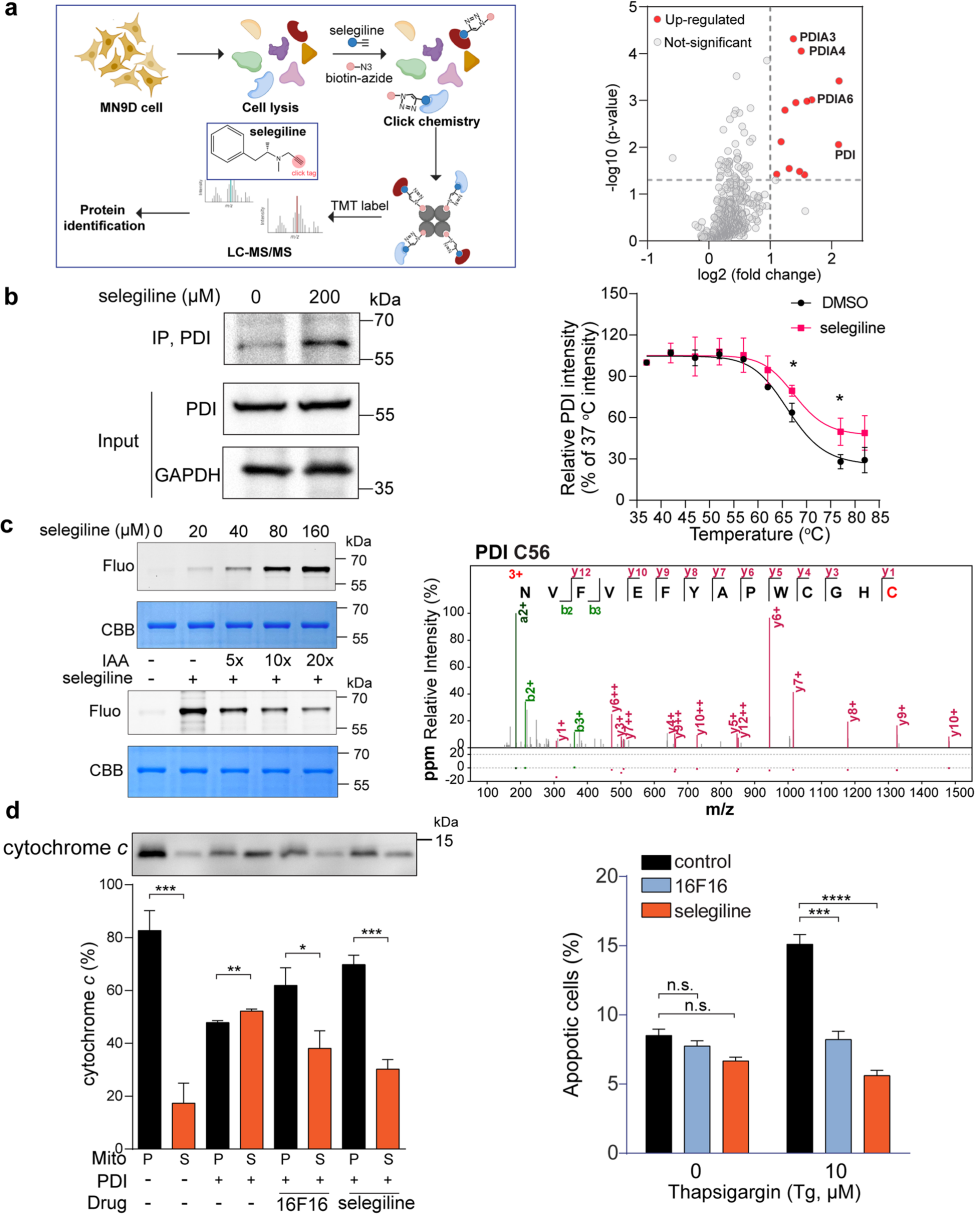
Selegiline binds to PDI and suppresses its pro-apoptotic activity. **a** Overall workflow of ABPP for the identification of the selegiline protein targets. The structure of selegiline is presented in the center. The clickable N-propargylamine moiety of selegiline is highlighted in red (Left). Scatter diagram representing proteins labeled by selegiline (100 μM) for 2 h in MN9D cell lysates compared to a DMSO treatment control. Fold change ≥ 2 and *p* value < 0.05 were considered as differentially expressed genes, which are highlighted in red (Right). **b** In situ pull-down assay verifying the interaction between selegiline and PDI. MN9D cells treated with 200 µM selegiline were ligated to biotin-azide and subjected to avidin pull down (Left). CETSA assessment of the binding between selegiline and PDI. MN9D cell lysates incubated with DMSO or 100 µM selegiline for 1 h were heated at designated temperatures, followed by western-blot analysis. The results were quantified and shown in the plot diagram (Right). **c** In-gel fluorescence visualization of recombinant human PDI labeled by selegiline in the presence or absence of IAA competition (Left). MS/MS spectrum of NVFVEFYAPWCGHC peptide from selegiline labeled PDI. The amino acid C highlighted in red indicates the cysteine 56 bound to selegiline (Right). **d** Immuno-blot and quantification of cytochrome *c* released from the MN9D mitochondria (pellet, P) into the supernatant (S) caused by PDI-triggered MOMP. Both PDI known inhibitor 16F16 and selegiline suppress the release of cytochrome *c* (Left). Quantification of apoptotic cells after 24 h of selegiline or 16F16 exposure, followed by 10 µM thapsigargin treatment for another 24 h (Right). Error bar, mean ± s.d. of *n* = 3 experiments. n.s., not significant; *, *p* < 0.05; **, *p* < 0.01; ***, *p* < 0.001; ****, *p* < 0.0001

PDI family members typically share thioredoxin (TRX)-like domains, which can be catalytic or non-catalytic. PDI contains four such domains (a, b, b’, a’), with a and a’ domains harboring a redox-active Cys-Gly-His-Cys (CGHC) motif that mediates its enzymatic activity [2]. C56, the selegiline modification site, is located within this catalytic motif. Thus, we next asked whether selegiline affects the enzymatic activity of PDI. We found that both selegiline and LOC14, a known PDI inhibitor, inhibited PDI reductase activity in an insulin aggregation assay (Supplementary Fig. 1f).

Despite the well-known role of PDI as a chaperone for disulfide bond modifications, recent studies have also revealed a pro-apoptotic function of PDI in neurodegenerative diseases [3]. For instance, misfolded huntingtin protein exposure leads to PDI accumulation at mitochondrial-associated ER membranes, triggering apoptosis via mitochondrial outer membrane permeabilization (MOMP) [4].To investigate whether selegiline's inhibition of PDI catalytic activity could rescue PDI-induced cell death, we isolated mitochondria from MN9D cells and conducted an MOMP assay with or without selegiline. As expected, purified PDI induced cytochrome *c* release, which was significantly suppressed by selegiline and the PDI inhibitor 16F16 (Fig. 1d, left). Given selegiline’s known MAO-B inhibitory properties, we also tested MAO-B's role in this process using pargyline, another MAO-B inhibitor. Pargyline did not inhibit PDI-induced cytochrome *c* release (Supplementary Fig. 1g), indicating that MAO-B is not involved. We then assessed selegiline's ability to rescue MOMP-triggered apoptosis. Pre-incubating MN9D cells with selegiline or 16F16 before inducing apoptosis with thapsigargin significantly reduced the percentage of apoptotic cells (Fig. 1d, right), highlighting selegiline’s potential to mitigate PDI-induced apoptosis.

While our current study provides valuable insights into the potential therapeutic effects of selegiline on PDI in PD, several limitations should be addressed. First, our findings only rely on the MN9D cellular model, which, though informative, does not fully capture the complexity of PD pathology. Future research should validate these findings in more diverse and physiologically relevant models. Previous studies by Hoffstrom et al. [4] and Kaplan et al*.* [5] have demonstrated the efficacy of PDI inhibitors in suppressing apoptosis in various models, including cells and brain slices. Aligning with these findings, our future work will expand to models such as induced pluripotent stem cell (iPSC)-derived neurons or PD animal models to gain a broader understanding of selegiline’s effects on PD pathology. Additionally, our current study did not systematically explore the dose–effect relationship of selegiline. Further investigations are needed to determine the optimal and physiologically relevant doses that exert therapeutic effects on PDI. Such studies would not only validate our current findings but also potentially broaden selegiline’s therapeutic applications.

In summary, we leveraged selegiline’s unique N-propargylamine moiety for ABPP and identified PDI as a key target in our study. Selegiline covalently binds to PDI at residues S32, C56, and K207, notably within the catalytic CGHC motif at C56, thereby inhibiting PDI's enzymatic activity. Typically, PDI plays a protective role in the unfolded protein response (UPR), but it can also exhibit pro-apoptotic functions in neurodegenerative diseases, leading to mitochondrial outer membrane permeabilization (MOMP) and cell death. Our findings reveal that selegiline-mediated inhibition of PDI prevents MOMP in isolated mitochondria and rescues ER stress-induced apoptosis in MN9D cells, thereby unveiling a novel aspect of its neuroprotective mechanism.

## Supplementary Information

Supplementary Material 1.

Supplementary Material 2.

Supplementary Material 3.

Supplementary Material 4.

## Data Availability

All data supporting the findings of this study are available upon request by contacting the corresponding author.
