# Supplementary figures and images for: A novel neuroprotective mechanism of selegiline by suppressing the pro-apoptotic activity of protein disulfide isomerase
Source: Mol Biomed. 2025 Mar 17;6:16. doi: 10.1186/s43556-025-00255-w (PMC11914425; doi:10.1186/s43556-025-00255-w)

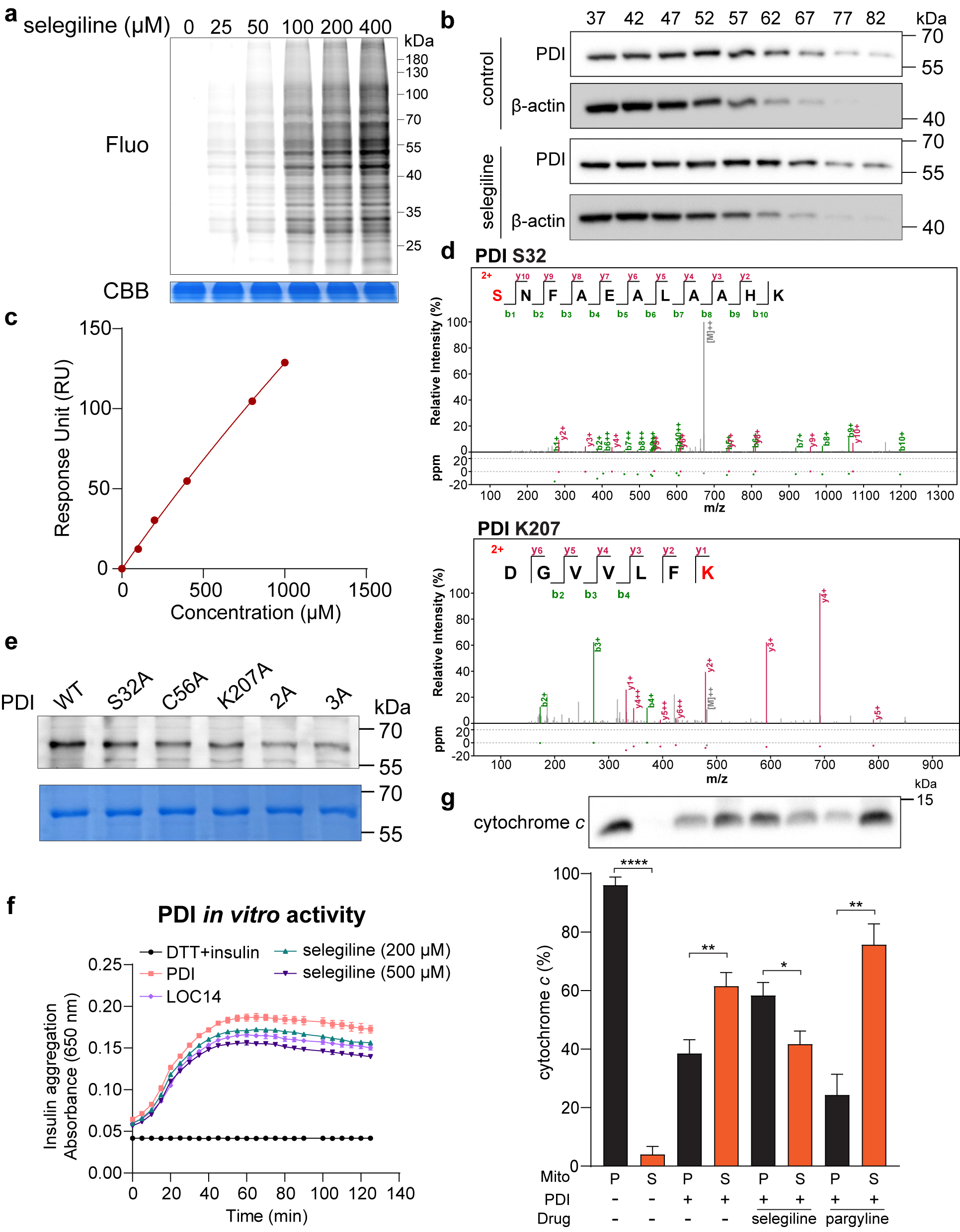

Supplement: Supplementary file 1 — Supplementary Material 1. [file 43556_2025_255_MOESM1_ESM.tif]

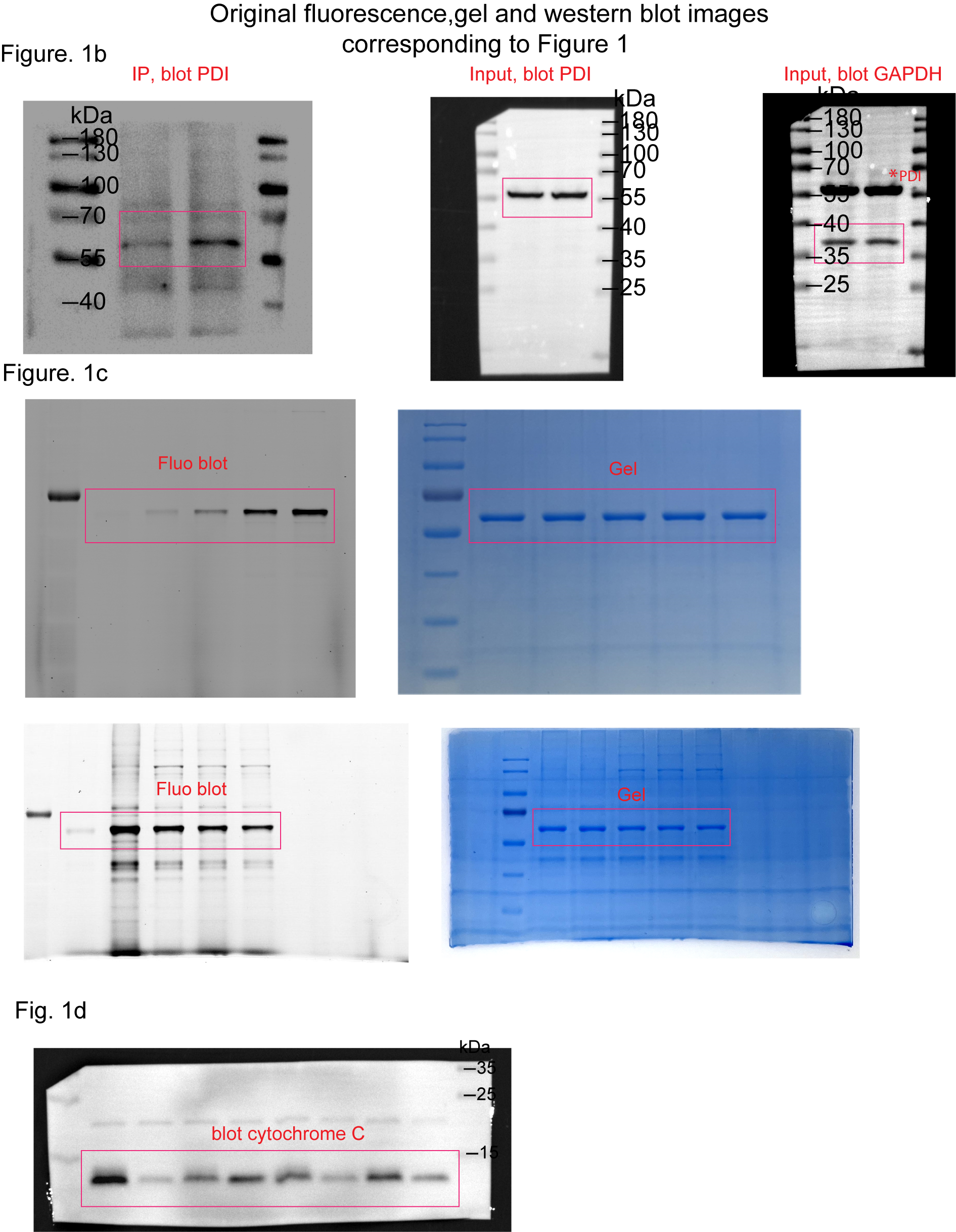

Supplement: Supplementary file 3 — Supplementary Material 3. [file 43556_2025_255_MOESM3_ESM.tif]

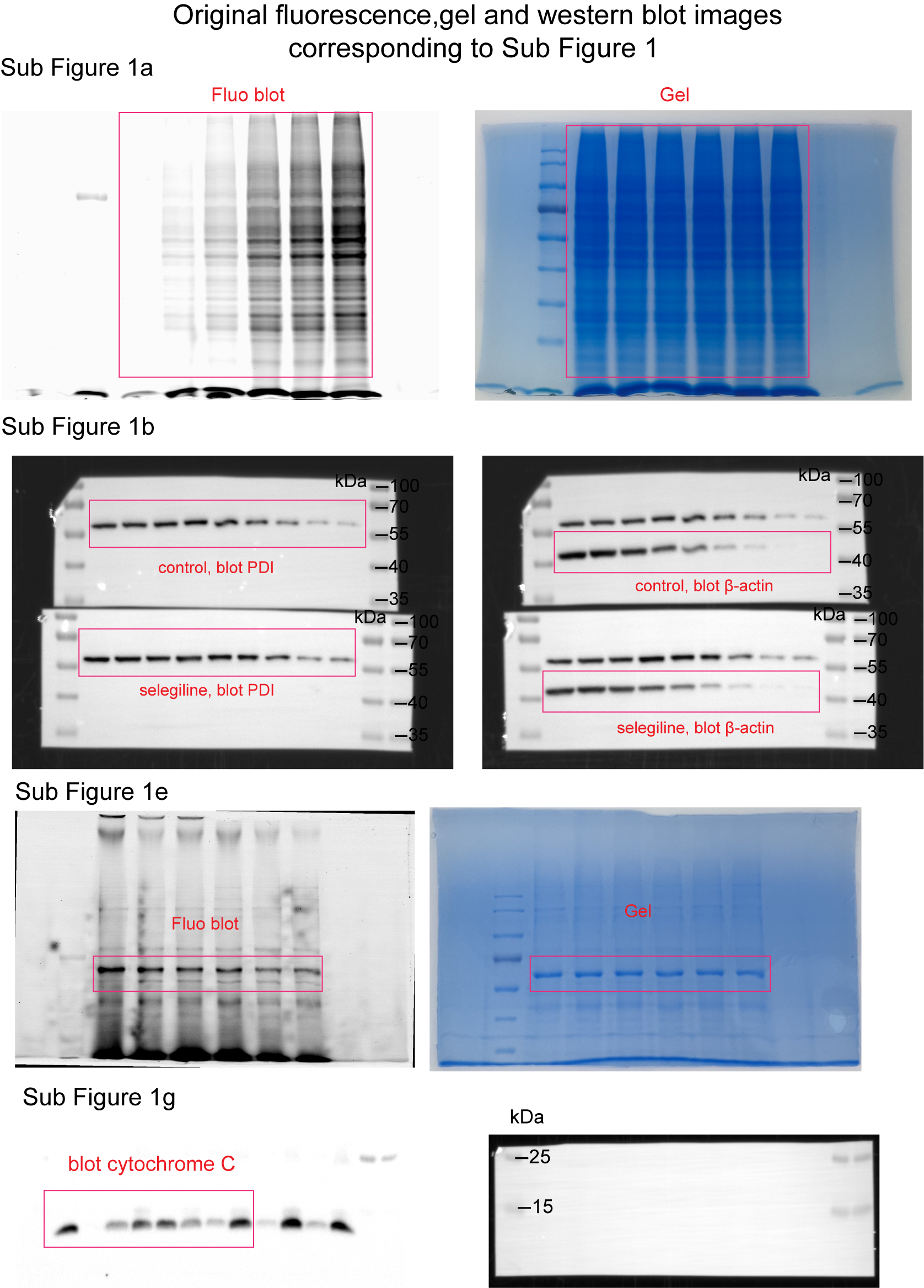

Supplement: Supplementary file 4 — Supplementary Material 4. [file 43556_2025_255_MOESM4_ESM.tif]
