# Supplementary material for: A novel neuroprotective mechanism of selegiline by suppressing the pro-apoptotic activity of protein disulfide isomerase
Source: Mol Biomed. 2025 Mar 17;6:16. doi: 10.1186/s43556-025-00255-w (PMC11914425; doi:10.1186/s43556-025-00255-w)
Supplement: Supplementary file 2 — Supplementary Material 2. [file 43556_2025_255_MOESM2_ESM.docx]

Supplementary Materials for

**A novel neuroprotective mechanism of selegiline by suppressing the pro-apoptotic activity of protein disulfide isomerase**

Yuting Xie^1,2†^, Bing Chen^1,2†^, Piao Luo^1^, Jingnan Huang^1^, Jigang Wang^1,2*^, Jichao Sun^1,2*^, Zhen Liang^1,2,3*^

^1^ Shenzhen Clinical Research Center for Geriatrics and Shenzhen Institute of Respiratory Disease, Shenzhen People’s Hospital (The Second Clinical Medical College, Jinan University; The First Afliated Hospital, Southern University of Science and Technology), Shenzhen, Guangdong, China.

^2^ School of Medicine, Southern University of Science and Technology, Shenzhen, Guangdong, China

^3^ Department of Geriatrics, Peking University Shenzhen Hospital, Shenzhen, Guangdong, China

^†^ These authors contributed equally: Yuting Xie, Bing Chen

^*^ Correspondence:

Zhen Liang:

liang.zhen@szhospital.com

Jichao Sun:

sunjichao@mail.sustech.edu.cn

Jigang Wang:

jgwang@icmm.ac.cn

**Materials and methods**

**Reagent**

Selegiline (#HY-14199) and pargyline (#HY-A0091A) were purchased from MedchemExpress (MCE, USA). Click chemistry reaction reagents including THTPA, TAMRA-azide and biotin-azide were from Click Chemistry Tools (CCT, USA); sodium ascorbate (NaVc) and CuSO_4_ were from Sigma-Aldrich (USA). High capacity neutravidin agarose resin, tetraethylammonium bromide (TEAB), sequencing grade modified trypsin, TMT^10^ plex reagent set and Pierce™ Quantitative Fluorometric Peptide Assay Kit were from Thermo Fisher Scientific (USA); Ni-NTA resin was from Smart-Lifesciences (China). PDI inhibitor LOC14 (#L288383) was from Aladdin (China) and 16F16 (#SML0021) was from Sigma-Aldrich (USA). Insulin (#PB180432) was from Procell (China). Annexin V-propidium iodide apoptosis detection kit (#KGA108) was from KeyGen Biotech (China). Rabbit polyclonal antibody against PDI (#ab137110) was from Abcam (UK); mouse monoclonal antibody against cytochrome *c* (#66264-1-Ig), GAPDH (#60004-1-Ig), rabbit polyclonal antibody against β-actin (#20536-1-AP), HRP-conjugated goat anti-mouse (#SA00001-1) and anti-rabbit IgG (#SA00001-2) were from Proteintech (China).

**In-gel fluorescence labeling assay**

Equal amounts of MN9D lysates were incubated with or without the selegiline for 2 h at room temperature. Cell lysates were subjected to click chemistry reaction with the click reaction cocktail (1 mM NaVc, 100 µM THPTA, 1 mM CuSO_4_, and 50 µM TAMRA-N3) for 1 h. Labeled proteins were precipitated using prechilled acetone. After sonication, dissolved proteins were mixed with SDS-PAGE sample buffer and resolved by SDS-PAGE gel. The fluorescence images were visualized using Sapphire Biomolecular Imager (Azure Biosystem, USA). For fluorescence labeling of recombinant PDI protein, purified PDI or PDI mutant proteins were incubated with selegiline at the indicated concentrations for 1 h at room temperature. For competition experiment, PDI proteins were incubated with IAA competitor for 1 h before selegiline treatment. Probe-labeled proteins were detected through a copper-catalyzed azide–alkyne cycloaddition reaction (CuAAC) with TAMRA-N3, followed by in-gel fluorescence analysis.

**ABPP-based targets identification**

MN9D cells lysates were treated with 100 µM selegiline for 2 h at 25 ℃. Click reagent was added to the lysates from a premix with 1 mM NaVc, 100 µM THPTA, 1 mM CuSO_4_, and 100 µM biotin-azide as the final concentrations, and incubated for 2 h at 25 ℃. After ice-cold acetone precipitation, clicked proteins were air-dried and resolubilised in PBS with 1.5% SDS. Protein samples were further diluted with PBS to a final concentration of 0.1% SDS and incubated with 50 µl pre-washed neutravidin agarose resin for 4 h at 25 ℃. Beads were washed 3 × with 1% SDS, 3 × with 0.1% SDS, 2 × with 6 M urea and 3 × with PBS. After the reduction with 10 mM DTT and the alkylation with 20 mM iodoacetamide (IAA), enriched proteins were digested with trypsin at 37 ℃ overnight with shaking. Peptides were loaded onto a pre-washed commercial C18 column for desalting, followed by labelling with TMT^10^ plex Mass Tag Labeling reagents. Target identification peptide samples were analyzed by LC‒MS/MS (Thermo Fisher, USA).

**Cellular thermal shift assay (CETSA)**

The CETSA experiment was performed as previously described [1,2]. Briefly, MN9D lysates were incubated with 100 µM selegiline or an equal volume of DMSO as a control for 1 hour at 25 °C with shaking. Cell lysates were divided equally and heated at designated temperatures for 3 min, followed by cooling on ice. The remaining soluble proteins were obtained by centrifugation at 20,000 × g for 20 min at 4 °C. Western-blot analysis was subsequently performed.

**Purification of His tagged PDI and PDI mutant proteins**

pCold II vectors encoding His-tagged human PDI wild type (WT) and PDI mutated variants were transformed into BL21 E. coli cells. After 0.3 mM isopropyl β-D-1-thiogalactopyranoside (IPTG) induction, bacterial cell pellets were re-suspended in lysis buffer (20 mM Tris-HCl pH 8.0, 200 mM NaCl, 1 mM PMSF, and 1 × protease inhibitor) and sonicated. Bacterial cell lysates were centrifuged at 12,000 × g for 30 min, the resulting supernatants were incubated with pre-washed Ni-NTA agarose resin for 2 h at 4 °C with constant rotation. Bead-immobilized proteins were washed twice each with lysis buffer containing 10 mM, 20 mM, and 50 mM imidazole. Lastly, lysis buffer containing 250 mM imidazole was used to elute proteins.

**Surface plasmon resonance (SPR)**

Surface plasmon resonance was performed as previously described [3], using a Biacore S200 SPR system (GE Healthcare, Sweden) at 25 °C. Purified recombinant PDI protein was immobilized onto a CM5 research-grade sensor chip via amine coupling. Selegiline solutions at concentration of 100 µM, 200 µM, 400 µM, 800 µM and 1 mM were flowed over the immobilized protein surface at 30 μl/min for 120 seconds, followed by a 300-second dissociation phase. Data analysis were conducted using the BioEvaluation software.

**Insulin aggregation-based PDI activity assay**

Insulin aggregation assay was performed as previously described [4] to detect the enzymatic activity of PDI, with slight modifications. Purified PDI proteins dissolved in reaction buffer (10 mM Tris-HCl, pH 8.0, 150 mM NaCl, and 2 mM EDTA) containing 100 μM bovine insulin were added to a 96-well black, clear-bottom plate. Wells were treated with either 0, 200, or 500 μM selegiline, or with 10 μM LOC14. DTT was added to each well to achieve a final concentration of 350 μM. The final reaction volume in all wells was 100 μL. The absorbance at 650 nm for each sample in the plate was read on a Tecan Infinite 200 microplate reader at 5-minute intervals for 2 hours.

**Mitochondrial outer membrane permeabilization (MOMP) assay**

MN9D cells were re-suspended in mitochondria extraction buffer (250 mM sucrose, 0.1% BSA, 10 mM Hepes pH 7.5, 5 mM KCl, 1.5 mM MgCl_2_, 1 mM EGTA, 1 mM EDTA) and incubated for 2 minutes on ice. Cell suspension was transferred to a Dounce Tissue Grinder (Sigma, USA) and homogenized on ice. After centrifugation at 700 × g for 10 min, supernatant containing the isolated mitochondria were combined and centrifuged at 10,000 × g for 10 min. Crude mitochondria in the pellet were re-suspended in mitochondria extraction buffer. Isolated mitochondria (50-100 ug per reaction) were incubated with purified PDI (16 µM) in the presence or absence of 16F16 (2 mM), selegiline (1 mM) or pargyline (1 mM) for 90 min at 37 °C with shaking. The mitochondria were then pelleted at 10,000 × g, 10 min at 4 °C. The degree of MOMP was calculated by western-blot analysis for cytochrome *c* released from the mitochondrial pellet into the supernatant.

**Cell death assay**

MN9D cells were treated with either 16F16 or selegiline for 24 h at 37 °C incubator, followed by incubation with 10 μM thapsigargin for another 24 h. The percentage of live cells was measured using flow cytometry analysis as described by the manufacturer.

**Statistics**

Data were analyzed using GraphPad Prism 8.0. The student’s *t* test was used to compare the means between two groups, and one-way ANOVA followed by Turkey’s test was used to compare the means among three or more groups. *p* < 0.05 was considered statistically significant.

**Reference**

1. Dai L, Zhao T, Bisteau X, Sun W, Prabhu N, Lim YT, et al. Modulation of Protein-Interaction States through the Cell Cycle. Cell. 2018;173(6):1481-94.e13. <https://doi.org/10.1016/j.cell.2018.03.065>

2. Jafari R, Almqvist H, Axelsson H, Ignatushchenko M, Lundbäck T, Nordlund P, et al. The cellular thermal shift assay for evaluating drug target interactions in cells. Nat Protoc. 2014;9(9):2100-22. <https://doi.org/10.1038/nprot.2014.138>

3. Luo P, Zhang Q, Zhong TY, Chen JY, Zhang JZ, Tian Y, et al. Celastrol mitigates inflammation in sepsis by inhibiting the PKM2-dependent Warburg effect. Mil Med Res. 2022;9(1):22. <https://doi.org/10.1186/s40779-022-00381-4>

4. Kaplan A, Gaschler MM, Dunn DE, Colligan R, Brown LM, Palmer AG, 3rd, et al. Small molecule-induced oxidation of protein disulfide isomerase is neuroprotective. Proc Natl Acad Sci U S A. 2015;112(17):E2245-52. <https://doi.org/10.1073/pnas.1500439112>

**Supplementary Figure 1**. **a** In-gel fluorescence (Fluo) visualization of MN9D proteome after labeling in vitro with indicated concentrations of selegiline for 2 h. The gel stained with coomassie brilliant blue (CBB) was shown below. **b** Western-blot analysis of CETSA assay conducted in Fig. 1b. β-actin was used as a loading control. **c** Surface plasmon resonance to assess the binding kinetic between selegiline and PDI protein. **d** MS/MS spectrum of SNFAEALAAHK and DGVVLFK peptides from selegiline labeled PDI. The amino acid S highlighted in red indicates the serine 32 bound to selegiline. The amino acid K highlighted in red indicates the lysine 207 bound to selegiline. **e** In-gel fluorescence visualization of recombinant WT and mutant PDI labeled with selegiline. 2A stands for mutation bearing S32A and C56A double mutants, 3A stands for mutation bearing S32A, C56A and K207A. **f** Insulin aggregation assay to measure the enzymatic activity of human recombinant PDI. LOC14 is a known PDI inhibitor and was used as a positive control (Right). **g** Immuno-blot and quantification of cytochrome *c* released from the MN9D mitochondria (pellet, P) into the supernatant (S) caused by PDI-triggered MOMP. Selegiline suppresses the release of cytochrome *c*, while MAO-B inhibitor pargyline does not. Error bar, mean ± s.d. of n=3 experiments. n.s., not significant; *, *p* < 0.05; **, *p* < 0.01; **** *p* < 0.0001.
